# “I am trapped in my body”: a qualitative exploration of bodily experiences during brace treatment among adolescents with idiopathic scoliosis

**DOI:** 10.1080/17482631.2025.2569615

**Published:** 2025-10-09

**Authors:** Marit Fure, Kristine Risum, Lena Fauske, Hedda Eik

**Affiliations:** a Section for Orthopaedic Rehabilitation Rikshospitalet, Division of Orthopaedic Surgery, Oslo University Hospital, Oslo, Norway; b Department of Rehabilitation Science and Health Technology, Oslo Metropolitan University, Oslo, Norway; c Department of Oncology, Norwegian Radium Hospital, Oslo University Hospital, Oslo, Norway; d Department of Interdisciplinary Sciences, Institute of Health and Society, University of Oslo, Oslo, Norway

**Keywords:** Adolescents, scoliosis, brace, musculoskeletal, qualitative research

## Abstract

**Purpose:**

Adolescent idiopathic scoliosis (AIS) is a spinal deformity that often requires long-term bracing to prevent the progression of spinal curvature. However, bracing impacts several aspects of adolescents’ lives. This study explores the bodily experiences of adolescents with AIS while receiving brace treatment.

**Methods:**

An exploratory qualitative study was conducted through in-depth interviews with 13 adolescents who had worn a brace for at least 6 months. The interviews explored adolescents’ experiences of scoliosis, and the brace’s impact on daily life. Data were analysed via reflexive thematic analysis informed by the phenomenological concept of embodiment.

**Results:**

Three main themes were identified in the analysis. First, the inhibited body, where adolescents described feeling restricted by the brace, which limiting physical activities and social interactions. Its rigidity hindered movement and participation. Second, the alienated body, where wearing a brace led to alienation and self-consciousness. Adolescents struggled with body image and feared negative feedback, and hid their brace under oversized clothing. Third, the disciplined body, where following bracing recommendations was difficult. Adolescents balanced required hours with social and school activities, often feeling guilt and frustration.

**Conclusions:**

A holistic approach is required to address the physical and emotional challenges of brace treatment and support adolescents’ overall well-being.

## 1. Introduction

Adolescent idiopathic scoliosis (AIS) is a spinal deformity characterised by lateral curvature and vertebral rotation (Weinstein, 2019). It typically occurs in otherwise healthy adolescents during the early stages of puberty (Cheng et al., 2015). AIS affects approximately 2–3% of the population, with 0.3–0.5% of those with AIS developing a magnitude of curvature that requires surgery (Negrini et al., 2018; Weinstein et al., 2008). The risk of curvature progression depends on the curve’s magnitude and the adolescent’s remaining growth. In this regard, curves reaching 40–50° post-growth are more likely to progress in adulthood, which increases the risk of spinal deformity, pain, functional limitations and reduced quality of life (Lonstein, 2006; Negrini et al., 2006). Moreover, adolescents with AIS often engage in less vigorous physical activity than their peers, potentially affecting their long-term health (Hannink et al., 2023). Conservative treatment for AIS aims to prevent curvature progression, respiratory dysfunction and pain while improving aesthetics through postural correction (Negrini et al., 2018). Bracing is effective in preventing progression into the surgical range (>50°) (Negrini et al., 2018; Weinstein et al., 2013), although its success is influenced by the level of adherence to wearing the brace (Dolan et al., 2020; Weinstein et al., 2013).

At Oslo University Hospital (OUH) in Norway, approximately 30–40 patients with Adolescent Idiopathic Scoliosis (AIS) undergo brace treatment each year, supported by a multidisciplinary team consisting of a physician, an orthotist, and a physiotherapist. Each rigid plastic brace, which extends from under the arms to below the hips, is custom-made to correct the curve and prevent the need for surgery. Two primary types of braces are commonly employed in the treatment of AIS: full-time braces, typically worn for up to 23 h/day, and nighttime braces, worn for approximately 8–10 h during sleep. Treatment choice is generally guided by the severity of the spinal curvature and anticipated patient adherence. The use of a brace can lead to adverse effects, including poor self-image, strained peer relationships, skin irritation, sleep disturbance and reduced physical activity (Dunn et al., 2018). These physical, emotional and social challenges often influence the patient’s treatment adherence (Rivett et al., 2009).

While a recent review found that patients with AIS generally report a good quality of life (QoL), it is often lower than that of peers without AIS (Essex et al., 2021). The predictors of reduced QoL include the scoliosis severity, perceived cosmetic deformity and depression levels, where patients with greater curvature report poorer outcomes (Kaya et al., 2022).

Research on AIS has often focused on surgical patients, particularly in qualitative studies examining self-image and appearance (Essex et al., 2022; Hannink et al., 2023). Existing studies on bracing tend to emphasize treatment efficacy, with less attention given to how adolescents experience the social and emotional aspects of wearing a brace. Nevertheless, most adolescents comply with brace treatment to avoid surgery and prevent curvature progression (Brigham & Armstrong, 2017).

Few studies have explored adolescents’ lived experiences of bracing. Yet, Cheung et al. (2022) identified obstacles such as physical discomfort and non-compliance with bracing, while Wang et al. (2022) highlighted fear of negative social feedback, physical discomfort and inconvenience as significant challenges with bracing.

Furthermore, Ghorbani et al. (2024) described brace adherence as a gradual process shaped by both internal motivation and external support systems. Their earlier work (2022) further underscored the practical and social challenges adolescents face in school settings, where brace use can interfere participation and peer interactions. Complementing these findings, Provost et al. (2006) proposed a model for interprofessional support that emphasizes collaborative strategies between healthcare providers and adolescents to strengthen treatment engagement.

Collectively, these studies highlight the complex and evolving nature of brace treatment in everyday life, providing an important backdrop for the present study. Our study aimed to extend the literature by exploring how adolescents with AIS experience changes in their bodily appearance and adolescence during brace treatment.

### 1.1. Theoretical framework

Phenomenology, which serves as both a theoretical framework and a method, emphasises the lived body (embodiment) as being central to meaning-making. This approach enables exploration of how adolescents experience the world as embodied beings through their thoughts, emotions, movements and perceptions within the dimensions of time and space (Merleau-Ponty, 2013).

Our aim was to uncover the deeper meanings associated with the lived body, guided by both intentionality and intersubjectivity. In this context, intentionality refers to consciousness’s inherent directedness towards objects or experiences, shaping individuals’ perceptions of their bodies and their lived experiences. Moreover, intersubjectivity highlights how experiences and knowledge are co-constructed through interactions, which are influenced by implicit, unspoken assumptions (Merleau-Ponty, 2013). Leder (1990) concepts of “dis-appearance” and “dys-appearance” deepen this understanding. Drawing on the work of Merleau-Ponty (2013) and Leder (1990) explained that when functioning well, the body recedes into the background (“dis-appearance”), being indirectly experienced through sensations supporting balance and awareness. Illness or discomfort disrupts this state, forcing the body into a state of awareness (“dys-appearance”). Leder (1990) also discussed “social dys-appearance”, where the body becomes objectified by others’ gaze, potentially causing alienation. Conversely, such awareness can foster acceptance and validation (Leder, 1990).

Both Merleau-Ponty (2013) and Leder (1990) revealed the dual role of the body as an invisible foundation for and visible obstacle to human experience. This framework provides valuable insights into the embodied experiences of adolescents with AIS who are undergoing brace treatment.

## 2. Methods

### 2.1. Participants and recruitment

We conducted an exploratory qualitative study through in-depth interviews with adolescents diagnosed with AIS and treated with a brace, focusing on their lived experiences from a phenomenological perspective (Kvale & Brinkmann, 2009). Purposive sampling (Braun & Clarke, 2019) was used to recruit participants from OUH’s brace outpatient clinic. The inclusion criteria were patients aged 12–15 years with AIS who were currently in brace treatment, who had worn a brace for at least 6 months, had no prior history of spinal surgical treatment, and who all spoke Norwegian. Adolescents aged 12–15 years were selected for inclusion, as this developmental window corresponds to the period during which brace treatment is most commonly initiated for individuals with adolescent idiopathic scoliosis (AIS). This age range typically coincides with peak growth velocity- a phase during which bracing is considered most effective- and thus reflects the clinical population most frequently targeted for non-operative intervention (Negrini et al., 2018, Sanders et al., 2008, Weinstein et al. 2013). Interviews were conducted until the data reached sufficient depth and variation to support meaningful thematic analysis, balancing saturation with richness rather than assuming exhaustiveness. Thirteen participants (three boys and ten girls) were interviewed. They had worn a brace for 6 months to 5 years: seven wore a full-time brace, three had transitioned from a full-time brace to a nighttime brace and three only wore a nighttime brace. All but one had participated in organised sports prior to the study. In this paper, the participants are identified using pseudonyms. The participants’ characteristics are detailed in Table 1.

### 2.2. Procedure

Participants were identified through OUH’s medical records and recruited by the first author, who contacted them by telephone prior to their scheduled consultation. They received written information about the study and were assured that neither participation in nor withdrawal from the study would not affect their treatment. All the invited participants agreed to take part and provided written informed consent, as did their parents.

The first author conducted face to face interviews in a meeting room during routine clinical follow-up at OUH between 12 November 2021 to 18 February 2022. The interviews lasted for an average of 33 min (range: 9–36 min, guided by a semi-structured interview guide (Supplementary file No. 1) exploring participants’ experiences of scoliosis, brace usage, interactions with healthcare professionals and feedback from friends and family. Emphasis was placed on the physical and social impacts of wearing a brace. Several participants provided concise yet insightful accounts, and the data proved sufficient richness for a meaningful thematic analysis**.** All the interviews were audiotaped, deidentified, transcribed and stored confidentially. This study was approved by OUH’s data protection officer (no. 21/20522). To ensure rigor of the study the Consolidated Criteria for Reporting Qualitative Studies (COREQ) was followed (Tong et al., 2007) and the checklist is available as Supplementary Material (Supplementary file No. 2).

### 2.3. Data analysis

We analysed the interview data via reflexive, thematic analysis, following Braun and Clarke (2019) guidelines for transparently identifying, analysing and reporting patterns. This reflexive approach was directed towards findings related to Merleau-Ponty (2013) and Leder (1990) theoretical concepts of the body. To fully comprehend the scope of the empirical dataset, the first, second and last authors reviewed all the interviews. The first author manually coded each interview during this empirical data analysis. Then, as a team, we discussed the codes, themes and subthemes. This collaborative effort resulted in three main themes depicting significant patterns relevant to our research question. In the final phase, the first and last authors drafted the analytical narrative, while the second and third authors provided feedback on multiple drafts. We identified compelling quotations from the transcripts that effectively illustrated our analytical points.

To ensure the rigor of the findings, we followed established principles for qualitative research quality, including credibility, dependability, and reflexivity (Korstjens & Moser, 2018). Credibility was supported through a transparent analytical process, researcher triangulation, and the use of verbatim quotes to ground interpretations in participants’ own words. Dependability was ensured by keeping an audit trail of analytic decisions and regularly revisiting the data throughout the coding process. The research team engaged in continuous reflexive discussions to identify and mitigate potential biases. In addition, preliminary findings were discussed within the team to test the consistency and robustness of the developed themes.

## 3. Results

Thirteen participants (three boys and ten girls) were interviewed. They had worn a brace for 6 months to 5 years: seven wore a full-time brace, three had transitioned from a full-time brace to a nighttime brace and three only wore a nighttime brace. All but one had participated in organised sports prior to the study. In this paper, the participants are identified using pseudonyms. The participants’ characteristics are detailed in Table 1.

All of the participants reported significant bodily changes after starting brace treatment, describing the experience of confronting a body they no longer recognised. This sense of disconnection was compounded by the negative attention directed not only towards the brace but also towards their altered body. As brace treatment typically coincides with adolescence, the participants often struggled to navigate both the physical challenges of the brace and the broader task of forming their emerging teenage identity. Balancing the treatment regimen with daily activities and responsibilities was repeated described as a challenge. Many participants reported difficulties meeting the demanding requirements of the brace treatment while maintaining their involvement in social interactions, school commitments and organised leisure activities. These challenges had ripple effects that disrupted multiple aspects of their daily life. Additionally, several participants expressed uncertainty about the treatment’s endpoint. They questioned whether they would achieve resolution using the brace or face the possibility of surgery in the future.

The participants’ experiences were encapsulated in three overarching themes: (1) the inhibited body, (2) the alienated body and (3) the disciplined body.

### 3.1. The inhibited body

Wearing a brace made the participants feel as though their body no longer cooperated as it did before. They described how the brace restricted their freedom of movement, making activities with friends, such as school trips and gym classes, more challenging. As Tuva explained, “Moving with a brace has been difficult for me, especially running—it just does not work. Physical activity and a brace are not a good combination. You cannot move, your back is stiff as a board, and you are not flexible at all”.

The rigid brace limited their movement, fostering a constant feeling of confinement. This sense of restriction was a persistent reminder of the brace’s presence. Previously, their body had blended seamlessly into their life, going unnoticed and being taken for granted. Now, they noted it to feel foreign and obstructive. The brace’s inflexibility restricted their participation in physical activities and ability to fully engage in social experiences with peers. Despite such challenges, healthcare professionals encouraged them to remain active and allowed them to remove the brace during physical activities, although doing so was not always practical. As Emma shared, “I wanted to dance because I loved performances. However, the breaks between performances were too long not to wear the brace but too short to keep taking it on and off. It became a hassle, so I eventually gave that up, too”.

The participants also reported ambivalence about not wearing the brace. Removing it offered physical relief but introduced new frustrations. Balancing activities and the need to wear the brace of a certain number of hours often felt impossible. Many struggled with the process of taking the brace on and off, frequently requiring adult assistance. This reliance on others proved particularly challenging at a time when they craved independence. Maintaining these routines became overwhelming, causing some participants to abandon activities they had once loved. Thus, the restricted body experience significantly impacted their daily life and sense of autonomy.

### 3.2. The alienated body

For many participants, wearing a brace fostered a profound sense of alienation from their body. Paradoxically, feeling “trapped” in their body also left them feeling “shut out” of their social world. They described adolescence as a time when belonging and acceptance are crucial, and the vulnerability associated with this stage of life amplifies the desire to fit in. As Anne explained, “I do not feel like a normal person when I wear it [the brace] […] weird, like I have something around me, that I am trapped in my body in a way”. This sense of entrapment translated into feelings of exclusion from peer communities. The participants feared standing out and longed for invisibility, hoping others would not notice their brace. Emma described this tension as follows: “Yes, it was just because I did not want to stand out. My biggest fear is to be known as 'the girl with back problems' or 'the girl who wears a brace'. I do not want to be known as that; I want to be known as 'Emma'. I started wearing oversized hoodies to hide it because I did not want questions; I did not want to stand out. So, it was tough starting middle school, and like all the girls started getting curves and showing off and all that, but I just wanted to cover up because I did not want to show that I had a brace”.

The participants distinguished between feeling different and looking different. The fear of being labelled, whether as sick, weak or abnormal, was deeply unsettling. Such labels could overshadow their identity, reducing them to their condition and eroding their social confidence. Many participants spent significant time hiding their brace with oversized clothing to avoid unwanted attention. Hence, they avoided wearing the clothes they wanted and instead chose outfits that would conceal the brace.

This persistent effort to appear “normal” reminded them of their perceived difference. For some, this vulnerability even shaped their relationships with peers. Trude shared the following: “I considered telling them when I started ninth grade, but I thought there was no point because I was already halfway through middle school. They have not noticed it much, as far as I know. It was difficult to tell them—I made four or five new friends then, and it was hard to tell them. And some I still have not told. […] If I explain it wrong, or if they reconsider whether they want to be my friend after all. […] that they will think it is weird”.

This alienation extended to romantic relationships, a key milestone in adolescence. As Peter reflected, “No, no, no! Everyone asks me about that [getting a girlfriend] because middle school is supposed to be the place where love starts, but it is not. Since I got the brace, I have not thought about girls; I must think about myself”. The brace created a dual sense of alienation. Their body felt foreign, not just because of the brace but because of how it set them apart from peers and their expectations regarding normal teenage life.

### 3.3. The disciplined body

The participants found it extremely challenging to follow the recommendations concerning wearing the brace, especially the required hours. They wanted to adhere to the guidelines but struggled with the sacrifices doing so entailed. Following the recommendations often meant forgoing activities integral to friends’ lives. As Anne explained, “All my friends talked about going to amusement parks and the beach, and I had to wear a 23-hour brace all day”. The participants described a daily process of negotiation to meet the time requirement of the brace treatment, weighing the benefits against the toll doing so took on their lives. Occasionally, they bent the rules, but this often led to guilt. Elin described this internal conflict as follows: “I want to have enough hours, so my back gets better quickly. And then, if I do not get enough hours, I am like, 'Oops, that was not so smart, but I could not get enough hours in'. One day, I’m slowly going to bed, and I’m like, 'Shit, now I will not get enough hours. I need to be back at school tomorrow'. So, I think I am delaying my recovery every time I do not get enough hours”.

The participants were forced to make adult decisions daily, an unusual burden at an age generally characterised by living in the moment. As their next medical check-up approached, many tightened their routines, sometimes adjusting the brace to improve the results. Pia expressed her worries as follows: “And I have been afraid to stop wearing the brace. I used to think it would finally be over when I stopped wearing it. […] However, I have thought about how things could get worse after the brace, like my back could get more crooked as time passes. And then I cannot do much about it. When I am done with the brace, there is not much more I can do, which scares me a bit […] Yeah, then it is too late. I have used my time; should I have used it better?” The participants experienced constant tension between immediate concerns and long-term outcomes. The brace forced them to navigate adult responsibilities while grappling with adolescent insecurities and social pressures. This struggle left them feeling both in control and powerless—not just over their spine but also over their life as a whole.

## 4. Discussion

The main findings of this study reveal how brace treatment for adolescents with AIS altered their appearance and life, creating a divide between who they were and who they want to be. The young participants expressed the need to appear normal, without standing out, while also desiring understanding from others about having a condition requiring care and follow-up. The brace, while necessary to treat their physical condition, also had profound psychological and social implications for them. Its rigidity restricted their freedom of movement and affected their ability to navigate an already challenging phase of life.

The participants reported a sense of bodily alienation, consistent with Leder (1990) concept of “dys-appearance”, where the body emerged as a source of discomfort and limitation. This alienation was compounded by social dys-appearance, as the adolescents feared revealing the brace and being defined by their condition. They described the fear of revealing the brace or being perceived as different to be a driving force behind their actions, such as avoiding social interactions and physical activities to protect their “normality”, echoing findings by Wang et al. (2022) and Ghorbani et al. (2022). Many feared being reduced to “a back” or “a person with scoliosis”, illustrating the dual impact of their internalised gaze and the perceived judgment of others. This alienation aligns with findings showing that puberty can evoke bodily estrangement, which is exacerbated by medical conditions (Carrasco & Solano-Ruiz, 2016). The adolescents in our study described how the brace made them negatively aware of their body, whereby the body was no longer experienced as a means of self-expression but rather as a source of limitation and difference. This reflects Merleau-Ponty's (1945) theory of bodily subjectivity, where the body shapes the individual’s identity and perception of the world.

Brace treatment inhibited the adolescents’ participation in physical activities, which is consistent with research showing reduced activity levels and increased sedentary time among adolescents with AIS (Chopra et al., 2020; Ghorbani et al. 2024; Newman et al., 2023). Our participants withdrew from activities that threatened their sense of “normality”, a coping mechanism that aligns with Sanderson et al. (2011) concept of the “struggle for normality”. Significant mental effort was dedicated to managing the participants’ feelings of difference, with their normalisation strategies including wearing oversized clothing to conceal the brace and asymmetric body shape. This finding is supported by other studies that note how adolescents with AIS use clothing as a form of camouflage (Carrasco & Solano-Ruiz, 2016; Hannink et al., 2023; Motyer et al., 2022). The visibility of the brace and how it deviates from societal norms concerning appearance haunted the participants daily. Brace usage was similarly linked to emotions such as embarrassment, stress, and fear of peer judgment, as reported by Ghorbani et al. (2024), reflecting patterns also observed in our study.

Having an appearance that deviates from “normal” was a common concern among the participants in our study, as the use of a rigid brace emphasised the visible asymmetry caused by their spinal curvature. The brace, designed to prevent curvature progression, often altered the wearer’s silhouette, making the asymmetry more noticeable. Moreover, the brace acted as a barrier to social integration, with the adolescents feeling stigmatised and defined by their medical equipment rather than by their personality or achievements. This reflects Goffman (1963) concept of “stigma management”, where individuals adopt strategies such as hiding or explaining their condition to minimise the stigma of being “different”. Such perceptions often lead to psychological stress, social isolation and a heightened sense of vulnerability (Rumsey & Harcourt, 2004), especially during a life stage where both physical appearance and social acceptance are critical.

In line with other studies involving youth with chronic illness, such as diabetes or juvenile idiopathic arthritis, the adolescents in our study described one of the most challenging aspects of brace treatment to be its intrusion into their daily life, where the demands of their medical follow-up often conflicted with their desire to live a typical teenage existence (Hagger et al., 2016; Min et al., 2022; Tong et al., 2012). Indeed, our participants faced a paradox: while adolescence is a time for independence, the brace treatment made them more reliant on parents and medical routines, exacerbating the feeling of lost autonomy (Essex et al., 2021).

The uncertainty about the outcome of their brace treatment undermined the adolescents’ confidence. This duality, between a sense of ownership and a feeling of imposed limitations, underscored the tension between control and autonomy in terms of managing a chronic condition during adolescence. The participants expressed concern about the long-term outcomes of their treatment, particularly the possibility of surgery. This uncertainty prompted discomfort and fear, consistent with findings by Toye et al. (2016). The brace was both a tool for empowerment and a reminder of limitations, reflecting the complexities of managing a visible and restrictive condition and its treatment during a formative stage of life.

## 5. Limitations

This study has several limitations. First, participants were included only if they had worn a brace for at least 6 months, excluding those in the early adjustment phase, a period that may be marked by emotional and social adaptation of the brace treatment. Still, this ensured that participants had sufficient experience to reflect meaningfully on the psychosocial impact of bracing. Second, the sample included both full-time and nighttime brace users, which may have shaped their experiences differently. Nevertheless, this variation contributed to a broader understanding of how adolescents navigate brace treatment across contexts. Third, interviews averaged 33 min (range: 9–36 min), and some were relatively brief, potentially limiting extended reflection. Even so, the conversations were focused and yielded nuanced insights into the complexity of living with a brace during adolescence.

## 6. Practical implications

These findings underscore the need for a coordinated, multidisciplinary effort to support adolescents throughout their bracing experience. Clinicians, orthotists, and educational personnel are uniquely positioned to address the psychosocial challenges associated with brace treatment. Enhancing communication between care providers and school staff, implementing individualized support strategies, and promoting psychosocial resilience may mitigate the emotional and social burden of treatment. Integrating such approaches into routine care could improve adherence, foster patient engagement, and contribute to more improved long-term outcomes.

## 7. Conclusion

This study highlights the profound impact of brace treatment on adolescents with AIS, which influences their daily life, social interactions, self-perception and coping strategies. The participants described how wearing a brace required intricate negotiations, such as concealing their brace with oversized clothing and modifying their behaviours to align with societal expectations and avoid stigma. The findings emphasise brace treatment’s emotional and social costs, including reduced participation in physical activities and the strain of relationships. The participants faced the dual challenge of adhering to medical recommendations and navigating the sacrifices required to maintain a sense of normalcy and engage in typical adolescent experiences. These findings underscore the importance of adopting a holistic approach to brace treatment that extends beyond physical management. Healthcare professionals, educators and policymakers must recognise and address the emotional and social challenges faced by adolescents with AIS, ensuring that support systems promote their overall well-being, self-esteem and QoL.

**Table 1.** Participant demographic and clinical information.

Gender
Female: 10
Male: 3
Age
12–15 years (median: 13.5 years)
Curve magnitude
25–45° (mean: 36°)
Brace use
Full-time: 7
Full-time/nighttime: 3
Nighttime only: 3
Brace models
Boston: 7 (3)
Providence: 2 (1)
Boston Night Shift: 2 (1)
Duration of brace use
0.5–5 years (mean: 1.96 years)
Full time 7 (range 0.5–5 years)
Night brace 3 (range 1–1.5 years)
Transition from full-time to nighttime bracing 3 (range 1–3.5 years)
Participation in organised sports
Yes: 12
No: 1

Note: Some participants used more than one brace model. The number in brackets includes those who transitioned between brace types.

## Supplementary Material

Supplementary material Supplementary_file_no_1_Interview_Guide

Supplementary material Supplementary_file_no_2_COREQ_checklist

## Data Availability

All of the data generated or analysed during this study are included in this published paper. The dataset generated and analyzed during the current study is not publicly available due to strict ethical regulation of health-related data in Norway. The consent to participate does not include permission to make the data available to a third party.
