# Supplementary material for: “I am trapped in my body”: a qualitative exploration of bodily experiences during brace treatment among adolescents with idiopathic scoliosis
Source: Int J Qual Stud Health Well-being. 2025 Oct 9;20(1):2569615. doi: 10.1080/17482631.2025.2569615 (PMC12517426; doi:10.1080/17482631.2025.2569615)
Supplement: Supplementary material — Supplementary_file_no_1_Interview_Guide [file ZQHW_A_2569615_SM6220.docx]

**Supplementary file no. 1 - Interview Guide for In-Depth Interviews in the Project: How Do Adolescents Experience Their Own Body During Brace Treatment?**

**🔹 Warm-Up Questions**

- Age
- Place of residence
- Activities (e.g., hobbies, sports)
- Type of brace
- How long have you been wearing a brace?

**🔹 Reflective Questions**

- Can you describe what it has been like to be a “brace-wearing adolescent”?
- Can you tell me about how it felt when you learned you had to start wearing a brace?
- How did you react when you were about to get your brace?
- How did you experience your encounter with the healthcare system and the information provided by the hospital about brace use?
- How open have you been about wearing a brace with those around you? What kind of experiences have you had?
- How have you felt about the way people around you have responded to your brace use? (friends, school, family?)
- Have you experienced anything positive about wearing a brace? For example, mastering the challenge of brace use?
- Have you experienced anything negative about wearing a brace? For example, negative comments, avoiding activities you would normally participate in?
- Many adolescents need to change braces several times due to growth. How has it been for you to experience your body changing during brace treatment?
- Have you had any problems with the brace? Has it broken, become too small, or caused other challenges?
- How has it been for you to meet others in the same situation?
- If you were to give advice to someone who is about to start wearing a brace, what would you say?

**🔹 Closing Questions**

- The road ahead - what will life be like without the brace?
- Is there anything you haven’t had the chance to say that you think I should know?
