# Supplementary material for: “I am trapped in my body”: a qualitative exploration of bodily experiences during brace treatment among adolescents with idiopathic scoliosis
Source: Int J Qual Stud Health Well-being. 2025 Oct 9;20(1):2569615. doi: 10.1080/17482631.2025.2569615 (PMC12517426; doi:10.1080/17482631.2025.2569615)
Supplement: Supplementary material — Supplementary_file_no_2_COREQ_checklist [file ZQHW_A_2569615_SM6221.docx]

**Supplementary file No 2.**

Consolidated criteria for reporting qualitative studies (COREQ): 32-item checklist

| **No** | **Item** | **Guide questions/description** | **Response** |
| --- | --- | --- | --- |
| **Domain 1: Research team and reflexivity** |  |  |  |
| Personal Characteristics |  |  |  |
| 1. | Interviewer/facilitator | Which author/s conducted the interview or focus group? | Marit Fure conducted the interviews |
| 2. | Credentials | What were the researcher's credentials? *E.g. PhD, MD* | MF: M Sc  KR: PhD  LF: PhD  HE: PhD |
| 3. | Occupation | What was their occupation at the time of the study? | MF: Researcher, PT  KR: Associate professor, PT  LF: Associate professor and researcher  HE: Associate professor. PT |
| 4. | Gender | Was the researcher male or female? | Female |
| 5. | Experience and training | What experience or training did the researcher have? | MF: Physiotherapist. Extensive clinical experience with scoliosis patients.  KR: Physiotherapist and researcher. Experience with clinical quantitative research.  LF: Qualitative research/patients’ perspective  HE: Physiotherapist and researcher. Extensive experience with qualitative research in health sciences  KR/LF/HE: Are all experienced supervisors for several master/PhD-students |
| Relationship with participants |  |  |  |
| 6. | Relationship established | Was a relationship established prior to study commencement? | Yes, MF/KR/HE  (KR and HE as supervisors) |
| 7. | Participant knowledge of the interviewer | What did the participants know about the researcher? e*.g. personal goals, reasons for doing the research* | The participants were told about the interviewers’ professional background, and the reason for conducting the study |
| 8. | Interviewer characteristics | What characteristics were reported about the interviewer/facilitator? e.g. *Bias, assumptions, reasons and interests in the research topic* | Experienced clinician  Well known with the scoliosis patient |
| **Domain 2: study design** |  |  |  |
| Theoretical framework |  |  |  |
| 9. | Methodological orientation and Theory | What methodological orientation was stated to underpin the study? *e.g. grounded theory, discourse analysis, ethnography, phenomenology, content analysis* | An explorative qualitative research design with a reflexive and interpretive phenomenological approach, and thematic analyzes underpinned the study. |
| Participant selection |  |  |  |
| 10. | Sampling | How were participants selected? *e.g. purposive, convenience, consecutive, snowball* | Participants were selected by MF on a purposive basis |
| 11. | Method of approach | How were participants approached? E*.g. face-to-face, telephone, mail, email* | Face to Face interview |
| 12. | Sample size | How many participants were in the study? | 13 participants |
| 13. | Non-participation | How many people refused to participate or dropped out? Reasons? | 1 participant was rejected due to age |
| Setting |  |  |  |
| 14. | Setting of data collection | Where was the data collected? e*.g. home, clinic, workplace* | Interview was collected at the hospital |
| 15. | Presence of non-participants | Was anyone else present besides the participants and researchers? | No |
| 16. | Description of sample | What are the important characteristics of the sample? *e.g. demographic data, date* | The study included ten girls and 3 boys, age 12-15 years of age. |
| Data collection |  |  |  |
| 17. | Interview guide | Were questions, prompts, guides provided by the authors? Was it pilot tested? | The interview guide was drawn on published literature in the field and the researchers clinical experience  No pilot |
| 18. | Repeat interviews | Were repeat interviews carried out? If yes, how many? | No |
| 19. | Audio/visual recording | Did the research use audio or visual recording to collect the data? | All interviews were audiotaped and transcribed |
| 20. | Field notes | Were field notes made during and/or after the interview or focus group? | Yes, field notes were made after the interviews |
| 21. | Duration | What was the duration of the interviews or focus group? | The interviews lasted 9-36 minutes |
| 22. | Data saturation | Was data saturation discussed? | It was assessed that the data had reached sufficient thematic depth when the final interviews no longer introduced new perspectives or topics. This suggests that the material provides a comprehensive understanding of the key experiences and reflections relevant to the study**.** |
| 23. | Transcripts returned | Were transcripts returned to participants for comment and/or correction? | No |
| **Domain 3: analysis and findings** |  |  |  |
| Data analysis |  |  |  |
| 24. | Number of data coders | How many data coders coded the data? | One, MF |
| 25. | Description of the coding tree | Did authors provide a description of the coding tree? | No |
| 26. | Derivation of themes | Were themes identified in advance or derived from the data? | Themes were derived from the data |
| 27. | Software | What software, if applicable, was used to manage the data? | No software used |
| 28. | Participant checking | Did participants provide feedback on the findings? | No |
| Reporting |  |  |  |
| 29. | Quotations presented | Were participant quotations presented to illustrate the themes / findings? Was each quotation identified? e*.g. participant number* | Yes, participant quotations were presented to illustrate the themes. A pseudonym was assigned to each participant to ensure their confidentiality. Neither gender nor age was linked to the quotes for the same reason. |
| 30. | Data and findings consistent | Was there consistency between the data presented and the findings? | There was consistency between the data, but also deviations within the material which gave us rich and nuanced examples suitable to illuminate the experiences from the participants’ own perspectives |
| 31. | Clarity of major themes | Were major themes clearly presented in the findings? | Yes, three major themes were presented |
| 32. | Clarity of minor themes | Is there a description of diverse cases or discussion of minor themes? | Yes, deviations within the material were presented |
